# Hematology and serum biochemistry of Indian spectacled cobra (*Naja naja*) and Indian rat snake (*Ptyas mucosa*)

**DOI:** 10.14202/vetworld.2016.909-914

**Published:** 2016-08-28

**Authors:** Sanath Krishna Muliya, Mudraje Narayana Bhat

**Affiliations:** Institute of Wildlife Veterinary Research, Karnataka Veterinary, Animal and Fisheries Sciences University, Doddaluvara - 571 232, Kodagu, Karnataka, India

**Keywords:** hematology, Indian rat snake, Indian spectacled cobra, *Naja naja*, *Ptyas mucosa*, serum biochemistry

## Abstract

**Aim:**

To study the hematology and serum biochemistry parameters of Indian spectacled cobra (Naja naja) and Indian rat snake (*Ptyas mucosa*) and to evaluate the differences in the same between captive and wild populations.

**Materials and Methods:**

Animals were categorized into four groups, *viz*., wild Indian spectacled cobra (n=10), wild Indian rat snakes (n=10), captive Indian spectacled cobra (n=10), and captive Indian rat snake (n=10). The snakes were restrained with restraint tubes, and 2 ml of blood was collected from either heart or ventral coccygeal vein. Hematological examinations were performed manually and serum biochemistry assays were performed on semi-automated clinical chemistry analyzer.

**Results:**

The values of total erythrocyte count, packed cell volume, and hemoglobin were slightly low in captive spectacled cobras and captive rat snakes compared to wild ones, whereas total leukocyte count was found to be slightly high in wild spectacled cobras compared to captive ones. All the recorded values of biochemical and electrolyte analytes were found to be well within expected range for snakes except for total protein and chloride levels in both the species which was slightly above the expected range.

**Conclusion:**

The hematology and serum biochemistry intervals of the two most common Indian snakes are presented here. The data will be useful in routine health evaluations and aiding in better medical management of the species studied. Since this study is the first to report complete hematologic and blood biochemical ranges for the study species, observations made here can also be used as referral intervals for future use.

## Introduction

The class Reptilia includes more than 10,000 extant species, which are segregated into four major orders. They are highly diverse, having biological and physiological peculiarities that differ both between and within major groups [1]. The Indian spectacled cobra (*Naja naja*) is elapid native to the Indian subcontinent which includes present day Nepal, Pakistan, India, Bangladesh, and Sri Lanka. Its distribution ranges from sea-level up to 2000 m (6560 ft) above sea-level [2]. Indian rat snake (*Ptyas mucosa*) is a colubrid found throughout South and Southeast Asia, from sea level up to 4000 m (13,120 ft). It is one of the most common snakes found throughout the country [3]. Both the species are often found in highly urbanized areas and settlements in the countryside, the attraction being the rodents commensal with man. Because of this, they are commonly involved in human-animal conflict. Cobras are usually killed or injured since they are venomous snakes. Indian rat snakes are capable of growling whenever they are threatened and more often than not; this snake is mistaken for animal such as the Indian spectacled cobra and gets killed because of this.

Numerous cases are presented frequently to wildlife veterinarians and wildlife rehabilitation centers throughout India, with the resultant injuries from such conflicts [4]. Captive populations of both the species are also found in almost all the Indian zoos. To prevent the predation and increase their chances of survival in nature, reptiles have evolved to be stoic, mastering the art of masking illness or disease, which makes them a challenge to diagnose and treat [5]. Very often, they require more than physical examination, a diagnostic tool such as hemato-biochemical analysis of blood, to arrive at a conclusive diagnosis [6]. In addition, with the increasing recognition of new diseases in reptiles and role of hematology and biochemistry investigations in diagnosing them, there is an urgent need to develop the evidence-based studies of disease and associated hematologic abnormalities in reptiles [7].

Without a doubt, one of the most useful interpretations of hematology is the change in either number or morphology of erythrocytes and lymphocytes that occur because of pathological conditions. Serum biochemical analysis is an equally important tool in evaluating the health of wild and captive animals. They help in understanding the functional ability of various organs, act as markers in diagnosing a pathological condition and in monitoring the psychological status of the captive as well as free-ranging snakes [8]. To interpret a laboratory report to be normal or abnormal, the values expected to be obtained from healthy animals (reference intervals) must be known first [9]. However, there is an acute paucity of hemato-biochemical studies on native reptiles in India. Most of the reference intervals available are from western countries, wherein the samples are sourced from captive animals living in altered environments that can significantly change the hemato-biochemical values. Hence, this study was carried out with an objective to establish reference intervals of hematological and biochemical parameters for two native snake species, the Indian spectacled cobra (*N. naja*) and Indian rat snake (*P. mucosa*).

## Materials and Methods

### Ethical approval

Necessary permissions were procured from Principle Chief Conservator of Forest (Wildlife), Karnataka to carry out the work.

### Study area and animals

The study was performed from August 2012 to April 2013 in Bengaluru, India. The wild, rescued Indian spectacled cobra (Group A1, n=10) and Indian rat snakes (Group B1, n=0) were procured from various wildlife rescue agencies and volunteers in and around Bengaluru, India, as soon as they were rescued and captive Indian spectacled cobra (Group A2, n=10) and Indian rat snake (Group B2, n=10) were procured from Bannerghatta Biological Park, India. Before being included in the study, each snake was subjected to a thorough physical examination, implemented in two parts: A hands-off examination and a hands-on examination. The hands-off examination was performed the first to get insight into snake’s general health, which included visual observation of respiration, behavior, and locomotion. During the hands-on examination, body condition was evaluated by assessment of epaxial muscles along with an estimation of parameters such as weight, sex, length, nutrition status, hydration, motility, and the presence of abnormalities. Only those snakes which appeared to be healthy and clinically stable based on above examinations, activity level, and body condition evaluation were selected for the study. Post blood collection, all the snakes were housed individually in plastic containers with hide boxes for 3 weeks to observe changes in activity and behavior.

### Blood collection and processing

The snakes were physically restrained with restraint tubes by one or two helpers. Blood (2 ml) was collected from either heart, or ventral coccygeal vein of the snakes depending on the size of animal. Snakes were placed in dorsal recumbency after restraining, and location of heart was determined by direct visualization of the beating heart or by manual palpation. Once located, it was isolated with the index finger and thumb. A 22-25 gauge needle attached to a 3 ml syringe was passed at approximately a 45° angle into the ventricle to collect the blood. In the case of larger snakes, the ventral coccygeal vein was used for venipuncture, wherein a 22-25 gauge needle attached to a 3 ml syringe was inserted along the ventral midline at a 45° angle between 2 ventral horizontal scales approximately 1/3 to 1/2 the distance from the vent. Whenever a clear fluid or diluted blood sample was obtained, the procedure was aborted and reattempted. All the procedures were carried out as quickly as possible with utmost care to minimize stress on snakes. Blood collection in rescued snakes was carried out within 1 h of rescuing so as to minimize the impact of capture stress on hematology and biochemistry parameters.

After collection, each sample was divided into a clot activator tube for serum biochemical analysis (1.5 ml) and lithium heparin collection tube for a complete blood count (0.5 ml). In addition, a direct smear of fresh blood was also made to carry out leukocyte differential counts. Whole blood sample collected in clot activator tube was allowed to clot for 30 min and later subjected to centrifugation at 3000 rpm for 30 min to separate serum. Both hematological and biochemical parameters were analyzed within 30 min of blood collection and serum separation, respectively.

### Blood analysis

Total erythrocyte count (TEC) and total leukocyte count (TLC) were determined with a 1:200 dilution of lithium heparin anticoagulated blood in Natt and Herrick’s solution [10,11]. Hemoglobin (Hb) was measured by cyanmethemoglobin method while packed cell volume (PCV) was calculated by microhematocrit method using capillary tube (3000 rpm for 30 min). Erythrocyte indices, namely mean corpuscular volume (MCV), mean corpuscular Hb (MCH), and MCH concentration were calculated using TEC, PCV, and Hb values. Each direct blood smear was stained with Giemsa’s stain and a 100-cell leukocyte differential count was performed to categorize cells into heterophils, monocytes, lymphocytes, eosinophils, or basophils [6].

Serum biochemistry assays were performed on semi-automated clinical chemistry analyzer (Labmate 10 Plus, Trivitron Healthcare Pvt. Ltd, Chennai, India) using commercially available biochemical kits (Transasia Bio-medicals Ltd. Himachal Pradesh, India), calibrated with control reagents before sample analysis. The analytes recorded included aspartate aminotransferase (AST), alanine aminotransferase (ALT), alkaline phosphatase (ALP), creatine kinase (CK), lactate dehydrogenase (LDH), blood urea nitrogen (BUN), creatinine (CRT), total protein (TP), and albumin (Alb). Serum electrolyte essay was performed on a compact electrolyte analyzer (LABLYTE™, Trivitron Healthcare Pvt. Ltd., Chennai) to analyze potassium (K), chloride (Cl), and sodium (Na).

### Statistical analysis

All recorded values were analyzed for statistically significant difference (p≤0.05) between the mean values of each group (*viz*., Group A1 and A2, Group B1 and B2) by student unpaired t-test, assuming unequal variance. All statistical analyses were performed using Microsoft office Excel version 2007 and results were interpreted as per standard procedures to arrive at conclusion [12].

## Results

Results of the hematology and serum biochemistry study in Indian spectacled cobra and Indian rat snake are tabulated in Tables-1 and 2, respectively. Quantity of blood collected (2 ml) from the individuals was sufficient to evaluate all the hematologic and serum biochemistry parameters. The values of TEC, PCV, and Hb were slightly low in captive spectacled cobras and captive rat snakes compared to wild ones and the TLC was found to be slightly high in wild spectacled cobras compared to captive ones. However, the differences in these values were statistically non-significant. MCV was the only parameter wherein a statically significant variation (p≤0.05) was seen between wild and captive Indian spectacled cobras.

**Table-1:** Hematology and biochemistry values observed for wild and captive Indian spectacled cobra (*N. naja*) with statistical data.

Parameter	Unit	N	Mean±SE		t-test	p value

			Wild, rescued Indian spectacled cobra (Group A1)	Captive Indian spectacled cobra (Group A2)		
TEC	×10^6^/µl	10	1.21±0.14	0.94±0.13	1.25	0.24
Hb	g/dl	10	8.23±0.39	7.68±0.25	1.11	0.29
PCV	%	10	33.10±1.89	31.10±2.32	0.72	0.49
MCV	fl	10	293.05±21.94	391.89±24.83	2.77	0.02^a^
MCH	pg	10	75.10±7.50	97.75±4.60	2.13	0.06
MCHC	g/dl	10	25.49±1.64	25.52±1.14	0.02	0.98
TLC	×10^3^/µl	10	10.26±0.61	8.67±0.72	1.47	0.18
Heterophils	%	10	45.60±4.05	48.70±3.02	0.70	0.50
Monocytes	%	10	0.70±0.34	0.50±0.40	0.35	0.74
Lymphocytes	%	10	52.30±4.00	49.10±3.07	0.65	0.53
Eosinophils	%	10	0.30±0.21	0.30±0.21	0.00	1.00
Basophils	%	10	1.20±0.79	1.40±0.60	0.19	0.86
LDH	IU/L	10	212.70±29.43	224.60±37.79	0.22	0.83
CK	IU/L	10	192.70±23.88	175.40±25.18	0.43	0.68
AST	IU/L	10	27.10±3.58	26.50±3.16	0.12	0.91
ALT	IU/L	10	4.70±0.73	5.70±0.83	0.88	0.40
ALP	IU/L	10	113.10±10.25	139.20±14.06	1.58	0.15
TP	g/dl	10	8.30±0.48	7.87±0.23	0.69	0.51
Albumin	g/dl	10	3.05±0.19	3.17±0.08	0.55	0.60
CRT	mg/dl	10	0.52±0.05	0.44±0.07	0.80	0.44
BUN	mg/dl	10	3.08±0.49	3.49±0.44	0.62	0.55
Potassium	mEq/L	10	5.18±0.37	4.69±0.28	0.91	0.38
Sodium	mEq/L	10	162.50±8.09	153.84±4.09	0.82	0.44
Chloride	mEq/L	10	133.12±3.75	136.58±2.43	0.70	0.50

a Statistically significant difference (p≤0.05) was observed between the mean values of MCV between Group A1 and Group A2. TEC=Total erythrocyte count, Hb=Hemoglobin, PCV=Packed cell volume, MCV=Mean corpuscular volume, MCH=Mean corpuscular hemoglobin, MCHC=Mean corpuscular hemoglobin concentration, TLC=Total leukocyte count, LDH=Lactate dehydrogenase, CK: Creatine kinase, AST=Aspartate aminotransferase, ALT=Alanine aminotransferase, ALP=Alkaline phosphatase, TP=Total protein, CRT=Creatinine, BUN=Blood urea nitrogen, *N. naja*=*Naja naja*

**Table-2:** Hematology and biochemistry values observed for wild and captive Indian rat snake (*P. mucosa*) with statistical data.

Parameter	Unit	N	Mean±SE		t-test	p value

			Wild, rescued Indian rat snake (Group B1)	Captive Indian rat snake (Group B2)		
TEC	×10^6^/µl	10	0.89±0.10	0.88±0.10	0.02	0.98
Hb	g/dl	10	7.95±0.27	8.22±0.44	0.50	0.63
PCV	%	10	36.30±1.27	34.20±1.92	2.11	0.06
MCV	Fl	10	444.19±36.47	433.50±54.59	0.14	0.89
MCH	Pg	10	100.68±11.84	104.31±11.99	0.22	0.83
MCHC	g/dl	10	22.18±1.15	24.84±2.32	1.21	0.26
TLC	×10^3^/µl	10	7.55±0.46	7.70±0.45	0.27	0.80
Heterophils	%	10	48.10±3.94	48.00±3.74	0.02	0.99
Monocytes	%	10	0.50±0.22	0.60±0.27	0.26	0.80
Lymphocytes	%	10	50.20±3.87	50.40±4.07	0.03	0.98
Eosinophils	%	10	0.20±0.13	0.10±0.10	0.56	0.59
Basophils	%	10	1.00±0.47	1.00±0.52	0.00	1.00
LDH	IU/L	10	199.70±40.18	289.20±44.57	1.36	0.21
CK	IU/L	10	280.40±41.80	200.50±24.96	1.43	0.19
AST	IU/L	10	77.80±13.25	72.40±14.07	0.24	0.82
ALT	IU/L	10	8.70±1.10	8.10±1.27	0.49	0.63
ALP	IU/L	10	111.20±10.72	85.80±11.78	1.53	0.16
TP	g/dl	10	8.24±0.30	7.65±0.36	1.21	0.26
Albumin	g/dl	10	3.11±0.10	3.13±0.12	0.13	0.90
Creatinine	mg/dl	10	0.39±0.08	0.45±0.06	0.53	0.61
BUN	mg/dl	10	3.03±0.40	2.85±0.32	0.36	0.73
Potassium	mEq/L	10	3.71±0.21	3.63±0.24	0.23	0.83
Sodium	mEq/L	10	169.24±5.54	162.11±6.12	0.71	0.50
Chloride	mEq/L	10	130.65±6.67	141.80±4.35	1.16	0.27

TEC=Total erythrocyte count, Hb=Hemoglobin, PCV=Packed cell volume, MCV=Mean corpuscular volume, MCH=Mean corpuscular hemoglobin, MCHC=Mean corpuscular hemoglobin concentration, TLC=Total leukocyte count, LDH=Lactate dehydrogenase, CK=Creatine kinase, AST=Aspartate aminotransferase, ALT=Alanine aminotransferase, ALP=Alkaline phosphatase, TP=Total protein, CRT=Creatinine, BUN=Blood urea nitrogen, *P. mucosa*=*Ptyas mucosa*

The variations in mean values of all the estimated serum biochemistry and electrolyte parameters between wild and captive snakes of both the species were statistically non-significant. In addition, all the recorded values of serum biochemical and electrolyte parameters were found to be well within normal range for snakes except for TP and chloride levels in both the species, which was slightly above the normal established range for reptiles (*viz*.; 3-7 g/dL and 100-130 mEq/L, respectively).

No notable change in activity and behavior was observed in any of the snakes until 3 weeks post blood collection. All wild snakes were released back uneventfully, and captive snakes were shifted back to their respective enclosures.

## Discussion

As an evolutionary response to avoid predation, reptiles have evolved to be stoic. This particularly makes them challenging to diagnose and treat. Hematologic evaluation in reptiles has thus gained increased importance in recent past as it can be used as to detect an array of pathological conditions such as anemia, inflammatory diseases, parasitemia, hematopoietic disorders, and hemostatic alterations [11]. Reptiles are reported to have lower TEC compared to mammals and birds, ranging from 300,000 to 2,500,000 erythrocytes/µL. Their PCV and Hb ranges from 20% to 40% and 6 to 10 g/dL, respectively [13-15]. Even though TEC, PCV, and Hb were slightly low in captive spectacled cobras and captive rat snakes compared to wild ones, they were in agreement with above findings. This variation observed between captive and wild snakes within the species may be attributed to extrinsic factors (season, temperature, environmental conditions, husbandry, diet, and living conditions) which are known to alter these parameters [11].

As tabulated in Table-3, hematologic values for Wild Indian spectacled cobra showed some similarities as well as differences when compared available values of other species of the genus *Naja*, namely Monocled cobras (*Naja kaouthia*), Siamese spitting cobras (*Naja siamensis*), and Golden spitting cobras (*Naja sumatrana*) [16]. The comparisons made were purely observational as statistical analyses were not feasible. A notable difference was observed in heterophil percentage of spectacled cobra (45.60±4.05) which was much higher than the reported values for Monocled cobras, Siamese spitting cobras and golden spitting cobras (4.4±1.0, 1.9±0.5 and 4.7±1.3). However, heterophil values in this study were found to be well within expected range for snakes [6,13]. Blood values for wild Indian rat snakes were not compared to any species as pursue of literature yielded no blood value reports from genus *Ptyas*.

**Table-3:** Comparison of hematologic values in snakes of genus *Naja.*

Parameter	Unit	Mean±SE			

		Wild Spectacled cobras^a^ (n=10)	Monocellate cobras^a^ (n=17)	Siamese spitting cobras^a^ (n=12)	Golden spitting cobras^a^ (n=6)
TEC	×10^6^/µl	1.21±0.14	0.616±0.052	0.576±0.042	0.657±0.086
Hb	g/dl	8.23±0.39	6.5±0.4	6.9±0.6	4.8±0.7
PCV	%	33.10±1.89	21.2±1.2	21.3±1.8	18.8±2.4
MCV	fl	293.05±21.93	362.7±18.9	371.6±24.9	289.3±9.6
MCH	pg	75.10±7.50	110.1±5.90	120.3±9.62	71.2±3.1
MCHC	g/dl	25.49±1.64	30.5±0.7	32.3±1.5	24.8±1.7
TLC	×10^3^/µl	10.26±0.61	14.316±1.265	12.025±0.880	9.816±1.046
Heterophils	%	45.60±4.05	4.4±1.0	1.9±0.5	4.7±1.3
Monocytes	%	0.70±0.34	1.2±0.4	1.0±0.5	0±0
Lymphocytes	%	52.30±4.00	66.9±4.4	71.8±3.5	69.2±4.1
Eosinophils	%	0.30±0.21	1.1±0.08	1.4±0.3	0±0
Basophils	%	1.20±0.77	0±0	0±0	0.2±0.2
Auzrophils	%	26.1±3.7	25.2±3.5	26.0±4.5

a From Salkij *et al*. [17] TEC=Total erythrocyte count, Hb=Hemoglobin, PCV=Packed cell volume, MCV=Mean corpuscular volume, MCH=Mean corpuscular hemoglobin, MCHC=Mean corpuscular hemoglobin concentration

The biochemical parameters are important to understand the functional status of individual organs and help understanding pathology of certain infectious diseases. However, the reference values for specific blood biochemical tests have been established for only a few of the described reptilian species [11,17,18]. Since environmental conditions, such as temperature, season, geographic area, ecological habitat, and wild versus captive status as well as physiologic factors such as species, nutritional status, reproductive status, gender, and age significantly affect the blood analytes of reptiles; it is important to have a region specific species referral interval that can help in interpreting general health status of concerned species [11,17].

Blood biochemical tests that appear to be most useful in reptilian diagnostics are TP, albumin, glucose, uric acid, AST, CK, and LDH. Normal reptilian AST level is usually <250 IU/L. LDH value in reptilian plasma is highly variable [19]. The AST and LDH activities are high in the liver tissue of reptiles and increase in their plasma activity may suggest hepatocellular disease [20]. The serum AST and LDH values observed in this study were well within the normal range for reptiles. Reptilian plasma ALT activity is usually <20 IU/L and not considered to be organ specific in reptiles [6]. The maximum plasma ALT level observed in present studies was 11 and 13 IU/L for Indian spectacled cobra and Indian rat snake, respectively.

The TP values observed in the present studies were slightly above normal established range for reptiles (3-7 g/dL). Studies suggest that TP above 7 g/dL is indicative of dehydration or chronic inflammation [6]. However, snakes studied in present work were apparently healthy, normally hydrated and were in visibly good body condition. BUN and CRT concentrations are generally poor indicators of renal disease in reptiles as terrestrial reptiles primarily being uricotelic, normal urea nitrogen concentration in these species is <10 mg/dL. According to earlier studies, even though CRT is a normal constituent of the urine of mammals, amount formed in most reptiles is negligible (<1 mg/dL) [6]. Observations made in present study correlate well with above facts as BUN and CRT value for wild as well as captive snakes of both species were <10 and <1 mg/dL, respectively. CK is considered to be a muscle-specific enzyme and can be used to detect muscle damage in reptiles. The maximum plasma CK level observed in present studies was 329 and 560 IU/L for Indian spectacled cobra and Indian rat snake, respectively. The values are highly variable in reptiles ranging up to 1500 IU/L [21].

Electrolyte levels in an animal give insight on various aspects such as hydration status, renal function, vitamin deficiencies, or various underlying diseases. Studies indicate that normal sodium level in reptiles ranges from 120 to 170 mEq/L and chloride level varies from 100 to 130 mEq/L [6,22]. The normal potassium level in snakes ranges from 3 to 6 mEq/L [23]. Chloride levels observed in this study were slightly higher than the normal range for reptiles. However, chloride concentration evaluation has no much significance in reptiles as it does not provide any clinically useful information [6]. The serum sodium and potassium values observed in present work were well within above-mentioned range for reptiles.

## Conclusion

The hematology and serum biochemistry intervals of the two most common Indian snakes have been presented here. The data will be useful in routine health evaluations, aiding in better medical management of the species studied. Since this study is the first to report complete hematologic and blood biochemical ranges for the study species, observations made here can also be used as referral intervals for future use.

## Authors’ Contributions

SKM and MNB conceived and designed the work. SKM conducted the experiment and analysis. Both SKM and MNB wrote and revised the manuscript. All authors read and approved the final manuscript.
